# Selection and Characterization of DNA Aptamers for Constructing Aptamer-AuNPs Colorimetric Method for Detection of AFM1

**DOI:** 10.3390/foods11121802

**Published:** 2022-06-18

**Authors:** Ruobing Liu, Fuyuan Zhang, Yaxin Sang, Minxuan Liu, Minghui Shi, Xianghong Wang

**Affiliations:** College of Food Science and Technology, Hebei Agricultural University, Baoding 071001, China; koori0520@163.com (R.L.); zhang.fuyuan@hotmail.com (F.Z.); sangyaxin@sina.com (Y.S.); lmx0325@126.com (M.L.); shiminghui0915@163.com (M.S.)

**Keywords:** aflatoxin M1, aptamer, MGO-SELEX, fluorescent assay, AuNPs

## Abstract

Aflatoxin M1 (AFM1), one of the most toxic mycotoxins, is a feed and food contaminant of global concern. To isolate the ssDNA aptamer of AFM1, synthesized magnetic graphene oxide nanomaterials, 12 rounds of subtractive systematic evolution of ligands by exponential enrichment (SELEX) selection were carried out. As a result, 24 candidate aptamers were selected, and their sequence similarity exceeded 97%. Their binding affinity and specificity were further examined by fluorescence and biofilm interferometry (BLI) methods. One aptamer (Apt-5) against AFM1 with a high affinity and specificity was isolated and demonstrated to be the optimal aptamer, whose dissociation constant reached the nanomolar level, Kd = 8.12 ± 1.51 nM. Additionally, molecular docking studies were used to predict the possible binding sites and mechanisms of the two. Based on Apt-5, an unlabeled aptamer-AuNPs colorimetric method was established to detect AFM1 in milk with a linear range of 0.078–10 ng/mL, and the actual detection limit was 0.078 ng/mL. These results demonstrated that this detection technique could be useful for the quantitative determination of AFM1 in milk and dairy products.

## 1. Introduction

Aflatoxin M1 (AFM1) is a metabolite formed by hydroxylation of aflatoxin B1 (AFB1) in the body after animals ingest AFB1. The toxicity is second only to AFB1. In 2012, International Agency for Research on Cancer (IARC) changed the classification of AFM1 from group 2 to group 1 to ensure safety for food and human health [1,2]. Once AFM1 is produced, it is difficult to eliminate because it is relatively stable to light, heat, and acid, which are the three most common methods of milk sterilization [3]. Excessive intake of AFM1 can damage tissues and organs, causing cancer, teratogenesis, mutagenesis, and even death [4,5]. At present, countries around the world have established strict limit standards for the content of AFM1 in food. The United States Food and Drug Administration (FDA) and the European Union (EU) set limits for AFM1 in milk to 0.5 ng/mL and 0.05 ng/mL, respectively [6,7]. There are many detection methods for AFM1. Although instrumental methods, such as gas chromatography and high-performance liquid chromatography [8,9], have the advantages of high sensitivity and strong stability, the complex equipment and tedious sample preparation limit their application in the rapid monitoring of AFM1 in the field [10].

Nucleic acid aptamers are single-stranded DNA or RNA that can specifically bind to the analyte, which is selected by systematic evolution of ligands by exponential enrichment (SELEX). They fold into complex and stable 3D structures, such as stem-loops, hairpins, and G-quadruplexes, through intermolecular forces, such as van der Waals forces, hydrogen bonds, and hydrophobic interactions, thereby achieving high-affinity and specific binding to target molecules [11,12]. Nucleic acid aptamers are also called “chemical antibodies”, and compared with antibodies, they are artificially synthesized with small batch-to-batch variation and have higher chemical stability, longer storage time, etc. [13,14]. In recent years, many research groups have screened and prepared aptamers for a variety of potential hazards in food matrices, such as biotoxins [15,16], pesticides [17], veterinary drugs [18], pathogenic microorganisms [19], and heavy metals [20]. As biological recognition components, aptamers have been widely used in the analysis of hazardous substances in food safety, showing outstanding detection performance, such as high sensitivity, simple operation, fast detection speed, and low cost.

Magnetic graphene oxide (MGO) composite nanomaterial is a combination of graphene oxide (GO) and Fe_3_O_4_ magnetic nanoparticles (Fe_3_O_4_/GO) [21,22]. It has excellent photoelectric properties, thermal stability, mechanical properties, superparamagnetism, and a large specific surface area [23,24]. At the same time, magnetic particles can improve the dispersibility of graphene oxide in water, and the large surface area of graphene oxide can support a large number of magnetic nanoparticles to prevent their agglomeration [25]. In this study, MGO combined with SELEX technology (MGO-SELEX) was used to screen high-affinity aptamers of AFM1, while MGO combined with molecular docking was used to predict and analyze the binding site and recognition mechanism. Using selected aptamers as specific recognition probes, the aptamer-AuNPs colorimetric method was established to effectively monitor the content of AFM1 in milk.

## 2. Materials and Methods

### 2.1. Materials

A single-strand DNA (ssDNA) library (5′-AGCAGCACAGAGGTCAGATG-40N-CCTATGCGTGCTACCGTGAA-3′) and primers (forward primer: 5′-AGCAGCACAGAGGTCAGATG-3′; reverse primer: 5′-TTCACGGTAGCACGCATAGG-3′; and phosphorylated reverse primer: 5′-P-TTCACGGTAGCACGCATAGG-3′) were synthesized by Sangon Biotech Co., Ltd. (Shanghai, China). AFM1, AFB1, Zearalenone (ZEN), Ochratoxin (OTA), Fumonisins (FB1), Deoxynivalenol (DON), and T-2 toxin were purchased from Yuanye Biological Technology Co., Ltd. (Shanghai, China), and 2 × Es Taq MasterMix (dye) was purchased from Kangwei Biotechnology Co., Ltd. (Beijing, China). The lambda exonuclease and 10 × lambda exonuclease reaction buffer was purchased from New England Biolabs Ltd. (Hitchin, UK).

### 2.2. Synthesis of Magnetic Graphene Oxide

The GO was synthesized from graphite powder by Hummer’s method [26]. MGO was prepared by the chemical coprecipitation method [27]. Briefly, 2 g FeCl_3_ and 1 g FeCl_2_ were added to 100 mL sterilized ultrapure water and mixed for 2 h; and 0.3 g GO was added to 100 mL sterilized ultrapure water, which was subjected to ultrasonic treatment for 4 h. Mix the above two solutions; add 30% ammonia water; adjust the pH of the solution to about 9~10; then heat to 90 °C while stirring until the color of the mixture was completely black. After cooling it to room temperature, it was centrifuged at 10,000 rpm for 5 min to obtain a black solid, which was washed several times with sterilized ultrapure water and absolute ethanol until the pH of the supernatant was about 7. Then, the black solid was dried in a drying oven at 70 °C; the dried solid was MGO.

### 2.3. In Vitro Selection of the DNA Aptamer

**Figure 1:**
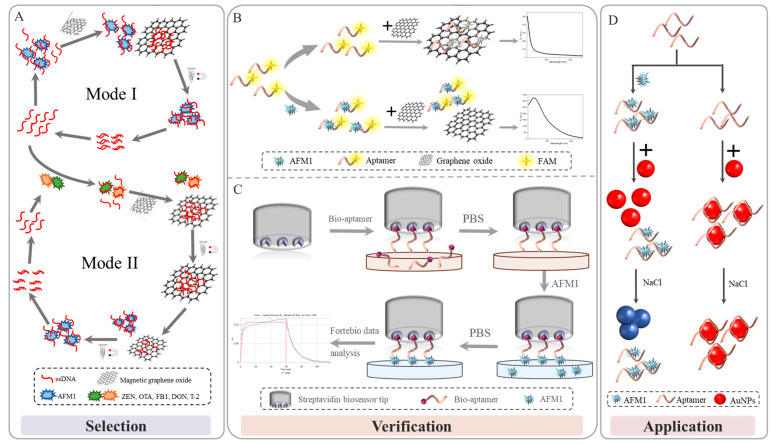
Schematic diagram (**A**) MGO-SELEX. (**B**) Fluorescence-affinity detection method. (**C**) BLI- affinity detection method. (**D**) Aptamer-AuNPs colorimetric method for detection of AFM1.

The procedure of MGO-SELEX of aptamer against AFM1 is illustrated in Figure 1A, following the protocol detailed in Table S1. Briefly, the ssDNA pool was heated to 95 °C for 5 min and allowed to cool naturally to room temperature to form the optimal structural conformation. The ssDNA library (1 nmol for initial round; 500–50 pmol for subsequent rounds) was incubated with AFM1 (The molar ratio to ssDNA library was 1:1) at 37 °C in 300 μL binding buffer (100 mmol/L NaCl, 20 mmol/L Tris-HCl, 2 mmol/L MgCl_2_, 5 mmol/L KCl, 1 mmol/L CaCl_2_, and pH = 7.4). Then, MGO suspension was added and incubated at 37 °C for 30 min. Under the action of an external magnetic field, the unbound ssDNA adsorbed on MGO was discarded, and the supernatant of the aptamer bound to AFM1 was used as a PCR template for amplification. The PCR reaction was performed in a 50 µL reaction buffer containing 2 μL ssDNA template, 1 μL forward primer (20 μM), 1 μL reverse primer (20 μM), 25 μL Mix, and 21 μL ddH_2_O. PCR conditions were as follows: 95 °C predenaturation for 5 min, followed by optimized cycles of denaturation at 95 °C for 30 s; annealing at 58 °C for 30 s; extension at 72 °C for 30 s; and a final extension step of 5 min at 72 °C. The next round of ssDNA library was prepared using lambda exonuclease-specific digestion phosphorylated antisense strands of PCR products; the digestion was conducted at 37 °C for 1 h. After being completely digested into ssDNA, the digestion mixtures were heated to 75 °C for 10 min to inactivate the enzyme. The digestion products were tracked by 8% denatured polyacrylamide gel electrophoresis. Finally, the ssDNA was purified by a phenol chloroform method and served as a nascent ssDNA pool for the next round of SELEX. Mode II counter-SELEX was employed at 6–10 selection rounds. For the counterselection, the denatured ssDNA pool was first incubated with ZEN, OTA, FB1, DON, and T-2 toxin (100 pmol individually) at 37 °C for 1 h; then, MGO was added and incubated at 37 °C for 1 h. Subsequently, MGO-ssDNA was then recovered, and AFM1 was added for positive selection as described above.

### 2.4. High-Throughput Sequencing

The enriched ssDNA in the last round was amplified by PCR using unmodified primers, following the same PCR conditions used for the SELEX process. The PCR products were subjected to high-throughput sequencing at Sangon Biotech Co., Ltd. (Shanghai, China) using Illumina sequencing technology. Use MEGAX 10.0.5 software (Auckland, New Zealand) to perform multiple sequence alignment and phylogenetic tree of the enriched sequences. The secondary structure and free energy of the enriched sequence were predicted by using the UNAFold Online Web server (http://www.unafold.org/, accessed on 31 May 2021). Based on the primary sequence, the secondary structures of the candidate aptamers, namely 24 candidate aptamers with more than 20 sequence repetitions in the high-throughput sequencing results, were selected for subsequent affinity and specificity analysis.

### 2.5. Characterization of Aptamer Binding Ability

To identify individual aptamer candidates, the quenching effect of the carboxyfluorescein (FAM), produced by graphene oxide (GO) through the adsorption of ssDNA, was used for the binding assay. Before the determination, the binding time of aptamer and AFM1, the mass ratio of GO and FAM-labeled aptamer, as well as quenching time and quenching temperature were optimized. Different concentrations (from 0 to 200 nM) of the fluorescence-labeled (5′-FAM) aptamer were allowed to react with a fixed amount of AFM1 (1 μM) in 200 μL of binding buffer in the dark for 1 h. Then, GO was added to the system, and the incubation continued for 15 min in the dark. The fluorescence spectrum of the final mixture under excitation at 492 nm was measured using an F-320 spectrofluorometer. Binding saturation curves and dissociation constants (Kd) were calculated by nonlinear fitting of relative fluorescence intensities using GraphPad Prism 9.0 software. The specificity of five sequences with lower Kd values (Apt-4, Apt-5, Apt-6, Apt-7, and Apt-10) was assessed using the same method. Then, 100 nM of aptamer was incubated separately with AFM1, AFB1, ZEN, OTA, FB1, DON, or T-2 toxin (200 nM individually) and set a blank control. The fluorescence values of the supernatants were measured.

In this study, four additional sequences (Apt-1, Apt-5, Apt-6, and Apt-10) were selected for binding affinity determination by BLI using the Octet RE96E system (ForteBio, Tianjin, China). All BLI measurements were performed at 25 °C after shaking at 1000 rpm in a 96-well plate containing 200 μL of binding buffer in each well. The aptamers were immobilized on the biosensor surface by the biotin–streptavidin coupling method between the modified biotin on the aptamers and streptavidin-coated biosensors. The immobilization process of aptamers was as follows: 200 μL of PBS (pH = 7.4); baseline (1 min); 200 μL of 1 μM biotin-aptamer (5 min); 200 μL of PBST (pH = 7.4); baseline (1 min). Then, the binding and dissociation within 1 min of the four sequences under 1 μM AFM1 were analyzed. The affinity parameter (Kd) was obtained using Octet Data Analysis Software (Sartorius, Göttingen, Germany) (Fortebio Data Analysis 10.0) after subtracting buffer and non-specific signals and after normalizing the response data, obtained from the reaction surface.

### 2.6. Molecular Modeling Studies and Truncated Design

To investigate the association mechanism between the developed aptamer (Apt-5) and AFM1, we used the Lamarckian genetic algorithm of AutoDock 4.2 software (Scripps Research, San Diego, CA, USA) for molecular docking. The Vienna number of the aptamer was formed through the UNAfold Online Web server, and then, the three-dimensional structure with the best-predicted energy was constructed using the RNA composer online tool. The three-dimensional conformation of the aptamer was obtained by mutating the U-base to the T-base. The structure files of the aptamer and AFM1 were operated by AutoDock Tools software to add H atoms, Gasteiger–Hücker empirical charge, merge non-polar hydrogen, and set rotatable bonds. During the docking process, the entire aptamer sequence was selected as the binding site, and AFM1 was independently docked at the binding site 200 times.

According to the predicted results and secondary structures, Apt-5 was truncated to obtain Apt-5T1, and Apt-5T2, while G27, A28, and T37 of Apt-5 were mutated to C to obtain Apt-5M. The sequence is shown in Table S1. After labeling the fluorescence, the affinity was determined by the method described in Section 2.5, and the fluorescence recovery was compared.

### 2.7. Aptamer-AuNPs Colorimetric Detection of AFM1

#### 2.7.1. Preparation of AuNPs

The preparation of gold nanoparticles (AuNPs) was carried out according to the previously reported method [28]. Specifically, 4.2 mL of 1% chloroauric acid solution was adjusted to 100 mL and heated to boiling; 10 mL of 1% sodium citrate solution was quickly added, and the boiling continued for 20 min under constant stirring. After cooling to room temperature, AuNPs solution was finally obtained.

#### 2.7.2. Method Feasibility

The stability of the AuNPs was evaluated by a colorimetric method. Briefly, 50 μL of AuNPs solution was incubated with AFM1 of different concentrations (0, 1.56, 3.12, 6.25, 12.5, 25, 50, and 100 ng/mL) for 30 min; then, the UV-vis spectra were acquired. Then, 100 mM NaCl (Final volume of 100 μL) was introduced, and the UV-vis absorption spectra were acquired; the extinction ratio at two wavelengths (Abs650/520) was used to reflect the aggregation degree for the AuNPs.

Then, 20 μL of 200 nM aptamer, 20 μL of AFM1, and 50 μL of AuNPs were immobilized in the system; the reaction sequence was adjusted, and their absorption spectra at Abs650/520 (Absorbance at 650 and 520 nm, respectively) were obtained. ssDNA library was used as the control for the aptamer.

#### 2.7.3. Optimization of Colorimetric Conditions

Different concentrations of NaCl (0, 7.5, 15, 30, 45, 60, 75, 90, and 105 mM) were mixed with 50 μL of AuNPs solution and incubated at 37 °C for 15 min (Final volume of 100 μL). The UV-vis spectra ranging from 400 nm to 700 nm were acquired.

Then, 20 μL of different concentrations of the aptamer (0, 50, 100, 200, 300, 400, and 500 nM) was mixed with 50 μL of AuNPs solution and incubated at 37 °C for 30 min; finally, 75 mM NaCl was added. The absorbance at 650 nm and 520 nm were measured, and calculated the ratio (Abs650/520).

Optimization study was conducted for two times in this study. One was performed for the incubation time of AuNPs and NaCl, which were, respectively, investigated for 1, 2, 3, 4, 6, 8, 10, 12, and 15 min. The other was the incubation time of aptamer and AuNPs, which were, respectively, investigated for 1, 2, 3, 4, 5, 7, and 10 min by fixing other conditions, and Abs650/520 were acquired.

#### 2.7.4. Colorimetric Detection of AFM1

In total, 20 μL of 200 nM aptamer was added to the same volume of AFM1 standard samples (0.078–10.0 ng/mL) of different concentrations and incubated at 37 °C for 1 h. Then, 50 μL AuNPs solution was added and incubated at 37 °C for 5 min. Finally, after adding NaCl, the color change was observed, and the Abs650/520 was acquired. A standard curve was generated using Origin 2021 software (Northampton, Massachusetts, MA, USA). To evaluate the specificity of the aptamer, the same procedures were followed as above with the samples of AFM1, AFB1, ZEN, OTA, FB1, DON, and T-2 toxin at 37 °C for 1 h and set a blank control at the same time.

#### 2.7.5. Real Sample Analysis

The milk samples were purchased from the local market of Hebei, China. First, the milk sample was centrifuged at 5000 rpm for 10 min, and the supernatant (Diluted 50 times with PBS) was used as a sample treatment solution. The sample treatment solution was added with different concentrations (0.1, 0.5, and 1 ng/mL) of AFM1, and the recovery rate was analyzed. A similar sample preparation procedure was used when analyzing AFM1 using a commercial ELISA kit.

## 3. Results and Discussion

### 3.1. Characterization of Magnetic Graphene Oxide

The morphology and nanostructure of MGO were characterized by a transmission electron microscope (TEM). It can be seen from Figure S1A that GO had an irregular sheet structure with a smooth surface and some wrinkles on it, which had the advantage of being a carrier. The spherical Fe_3_O_4_ had a particle size of 10–20 nm and was uniformly attached to the surface of GO. Meanwhile, the composite of Fe_3_O_4_ with GO can improve the aggregation of Fe_3_O_4_ NPs. The magnetic strength of MGO was characterized by a vibrating sample magnetometer (VSM), as shown by the hysteresis loop in Figure S1B; the curve was symmetrical, and smooth and the saturation magnetization was 66.15 emu·g^−1^, indicating that the MGO exhibited superparamagnetism and high magnetic saturation. It showed that MGO had a strong response ability to external magnetic fields, which would realize solid–liquid separation and recovery.

### 3.2. Selection of Aptamers for AFM1

MGO-SELEX technology has the characteristics of GO strong adsorbing non-specific ssDNA through hydrophobic interaction and π–π stacking interaction, which retains the advantage of easy separation of magnetic materials. At the same time, it avoids the coupling process of common magnetic and target materials and the steric hindrance effect caused by it so as to achieve the effect of efficient separation [29]. In order to compare the adsorption capacity of MGO and GO on ssDNA, different mass ratios and reaction times were set for incubation. The results are shown in Figure S1C,D. When MGO: ssDNA ratio was 50:1, the adsorption capacity was equivalent to when GO: ssDNA ratio was 200:1. When the reaction time reached 20 min, the concentration of ssDNA in the supernatant decreased significantly, but GO desorbed slightly with the increase in adsorption time. MGO had a stronger adsorption capacity and stability than GO, which might be due to the integration of Fe_3_O_4_^,^ making it a larger specific surface area. Although the mass ratio of MGO to ssDNA was 50:1, showing a strong adsorption capacity, in order to ensure complete adsorption and avoid non-specific dissociation, a ratio of 100:1 was chosen for a more stable experimental condition. Similarly, to ensure stable adsorption, 30 min was determined as the adsorption time of MGO (Figure S1D). In total, 12 rounds of MGO-SELEX were executed. To select AFM1 aptamers with high affinity, the concentration of the library and the time with AFM1 gradually declined during the selection in this work. As shown in Figure 1A, during the first five rounds, Mode I was employed to remove unbound DNA from the AFM1 via π–π stacking interactions between MGO and free ssDNA. Mode II was performed in the sixth round to improve the binding specificity; the ssDNA sequence that can bind to other mycotoxins ZEN, OTA, FB1, DON, and T-2 toxins were eliminated. This counter-SELEX greatly improved the screening efficiency [30]. Mode II was based on the high affinity of selected aptamers to induce desorption of ssDNA from MGO. No significant decrease in recovery ratio was observed even under highly stringent conditions. Subsequently, two rounds of Mode I screening were carried out, and the recovery rate was stabilized. Therefore, the selection cycles were stopped, and Pool 12 was amplified for high-throughput sequencing (HTS).

### 3.3. Binding Properties of Individual Aptamer to AFM1

Tens of thousands of sequences were obtained after high-throughput sequencing. Twenty-four candidate aptamer sequences with more than 20 sequence repetitions were selected for binding affinity analysis with up to 97% homology. As shown in Figure 1B, GO can physically adsorb aptamers labeled with fluorescent dyes, triggering fluorescence resonance energy transfer (FRET), and the fluorescence was quenched. Upon addition of AFM1, the aptamer was bound to AFM1 and was desorbed from the GO surface, resulting in recovered fluorescence. The aptamer was reacted with AFM1 for 1 h at 37 °C to reach equilibrium (Figure S2A). When the mass ratio of GO to FAM-labeled aptamer was 20:1, the fluorescence was quenched by 90%. As the ratio increased, the fluorescence intensity remained stable. Therefore, the ratio of 20:1 was selected for the measurement. The fluorescence intensity was the lowest when the quenching time was 15 min (Figure S2B). As time goes by, the slight increase in fluorescence intensity might be due to the dissociation of a small part of the aptamer; therefore, 15 min was selected as the quenching time (Figure S2C). Under the premise of the combination of aptamer and AFM1, the highest fluorescence intensity was seen when the reaction temperature was 37 °C (Figure S2D). By measuring the fluorescence intensity of the bound aptamer under optimal conditions, a nonlinear fitting curve was generated, and the Kd value of the aptamer sequence was calculated. These sequences and Kd values are summarized in Table S1, and the nonlinear fitting curve of affinity is summarized in Figure S3.

**Figure 2:**
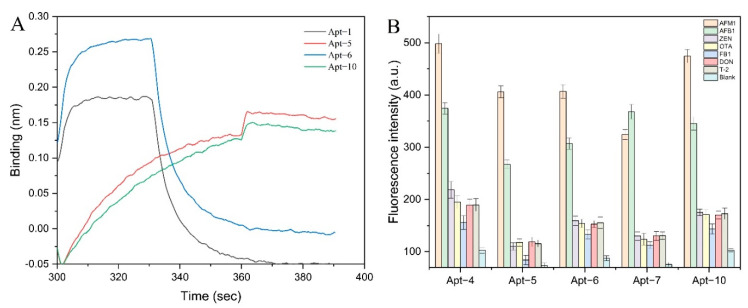
(**A**) The result of BLI determining the affinity of the aptamers. (**B**) Specific characterization of aptamers.

BLI is a label-free and real-time optical analytical technique that uses fiber-optic biosensors to measure biomolecular interactions. As shown in Figure 1C, the visible light emitted by the spectrometer forms a beam at the two interfaces of the optical film layer at the end of the sensor to form an interference spectrum. The thickness and density of the formed film vary due to the binding or dissociation of the biorecognition molecules immobilized on the surface of the biosensor tip with the analyte in solution. It is reflected by the displacement value of the interference spectrum, and a real-time response monitoring map is made through this displacement value [31,32,33]. The affinity detection result of the BLI method is shown in Figure 2A. The binding and dissociation within 1 min of the four sequences under 1 μM AFM1 were analyzed, and the Kd values were shown. The combined fluorescence results showed that although the Kd values were orders of magnitude difference, they all showed that Apt-5 and AFM1 interacted with high binding affinity. BLI provided a convenient and fast means for determining the extent to which AFM1 and aptamer can interact, as well as for quantifying the affinity.

Based on the results of affinity analysis, aptamers Apt-4, Apt-5, Apt-6, Apt-7, and Apt-10 with the lowest Kd value (i.e., the highest binding affinity) to AFM1 were selected for specificity analysis. As displayed in Figure 2B, in contrast, Apt-5 had a lower fluorescence intensity than other negative controls. Therefore, it was clear that Apt-5 had an excellent binding affinity and specificity for AFM1.

### 3.4. Study on the Binding Mechanism between Aptamer Apt-5 and AFM1

**Figure 3:**
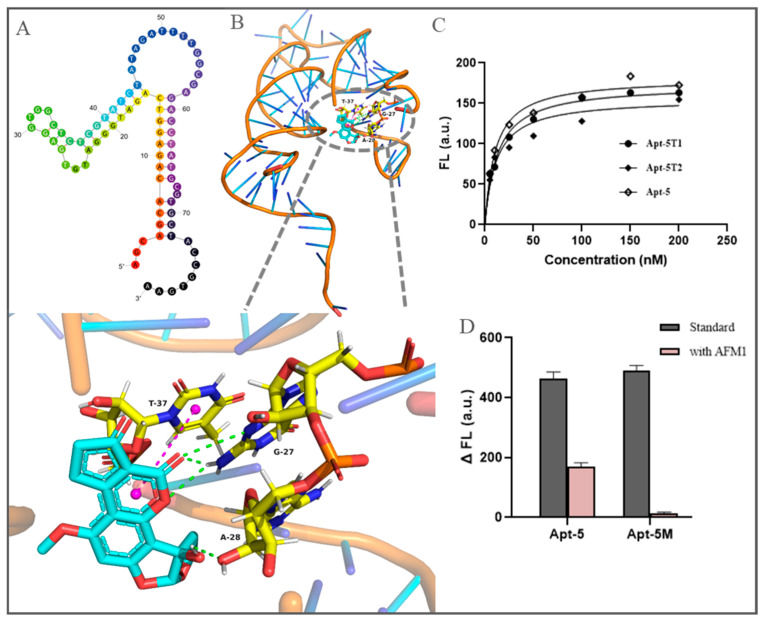
(**A**) Molecular docking results depicting interaction between aptamer Apt-5 and AFM1 (the green dashed line represents hydrogen bonding, and the pink dashed line represents hydrophobic interaction). (**B**) Secondary structure of Apt-5. (**C**) Nonlinear fitting curves of truncated sequences Apt-5T1, Apt-5T2, and full-length Apt-5. (**D**) Fluorescence recovery of Apt-5 and mutant sequence Apt-5M.

The binding mechanism and possible binding sites of the aptamer-AFM1 complex were further studied by molecular docking. The binding confirmation with the best docking score from the optimal clustering was selected. In Figure 3B, it is clearly seen that four hydrogen bonds and hydrophobic interactions play a major role in the formation of the aptamer Apt-5 and AFM1 complex. The main binding sites of aptamers are the formation of hydrogen bonds between G27, A28, and AFM1 and the hydrophobic interaction between T37 and AFM1’s benzene ring. Combined with the secondary structure of the aptamer, it is found that this site is exactly in the double-loop hairpin structure formed by G20-T40.

According to the predicted results of molecular docking, combined with the secondary structure of Apt-5, we carried out a truncated design. The two truncated sequences, Apt-5T1 and Apt-5T2, were 53 and 21 bases, respectively, and both retained the stem-loop structure, where G27, A28, and T37 were located. Affinities were comparable when compared to full-length sequences (Figure 3C). Additionally, we obtained Apt-5M by mutating G27, A28, and T37 to C. As displayed in Figure 3D, it was found that the fluorescence intensity of Apt-5M did not recover significantly after the reaction with AFM1. Such changes made its binding ability disappear, possibly because the base mutation destroyed the original stem-loop structure. Therefore, this region was the potential area for binding.

### 3.5. Colorimetric Detection of AFM1

The aptamer can be adsorbed to the surface of AuNPs due to electrostatic interaction so that it can remain dispersed in the salt solution without changing its color. In the presence of AFM1, the aptamer specifically binds to it, and the AuNPs, after the loss of protection, aggregate under the action of salt solution, with the color changing from red to blue. Based on this, quantitative detection of AFM1 can be achieved [34,35]; the principle is shown in Figure 1D. The AuNPs solution prepared in this experiment was wine-red; the solution was transparent and uniformly dispersed; there were no floats and precipitates. The UV-vis absorption spectrum of the AuNPs solution was scanned at 400–700 nm, while the size and shape of the AuNPs were measured by a transmission electron microscope. It can be seen from Figure S4A that the prepared AuNPs solution has a maximum absorption peak at a wavelength of 526 nm; the absorption peak is sharp; no impurity peaks appear, indicating that the prepared AuNPs solution has a uniform particle size. Additionally, the TEM showed that the size of the AuNPs particles was basically the same; there were no elliptical or polygonal AuNPs particles, and the average particle size of the AuNPs was 15 nm (Figure S4B).

#### 3.5.1. Feasibility Analysis

**Figure 4:**
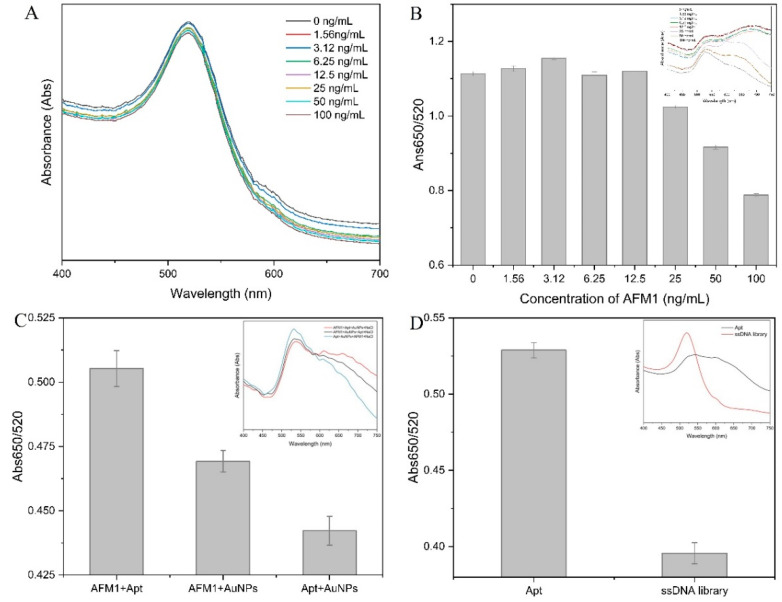
UV-vis spectra of AuNPs in the absence (**A**) and presence (**B**) of NaCl in the presence of different concentrations of AFM1. (**C**) The effect of AFM1, aptamer, and AuNPs reaction sequence. (**D**) Comparison of binding ability of aptamer and ssDNA library.

We first measured the colloidal stability of the AuNPs. It can be seen from the UV spectrum in Figure 4A that the characteristic peaks of AuNPs did not change significantly when the AFM1 concentration increased from 0 to 100 ng/mL, indicating that AFM1 did not cause the aggregation of AuNPs. We then demonstrated that some aggregation occurred in all samples by adding a final concentration of 100 mM NaCl to the above samples (Figure 4B). Interestingly, however, we noticed that AFM1 appeared to be slightly inhibited in the presence of high concentrations of aggregation, and the absorbance ratio was reduced compared to low concentrations. Similar to a previous report [36], we speculated that AFM1 would also be slightly adsorbed on AuNPs. We adjusted the order of adding AFM1, AuNPs, and aptamers to avoid the effect of AuNPs on the adsorption of AFM1. It can be found that in case of the same amount and same reaction time, the first reaction of AFM1 with AuNPs will inhibit the binding of aptamers to the target to a certain extent, and AFM1 cannot displace pre-adsorbed aptamers from AuNPs. Therefore, the pre-binding of the target and the aptamer can better reduce the error caused by the adsorption of AuNPs. Furthermore, we demonstrated the feasibility of the established label-free colorimetric assay based on the screened aptamers and AuNPs to detect AFM1 by using an equal amount of the initial random library as a control sequence for aptamers.

#### 3.5.2. Optimization of Colorimetric Conditions

**Figure 5:**
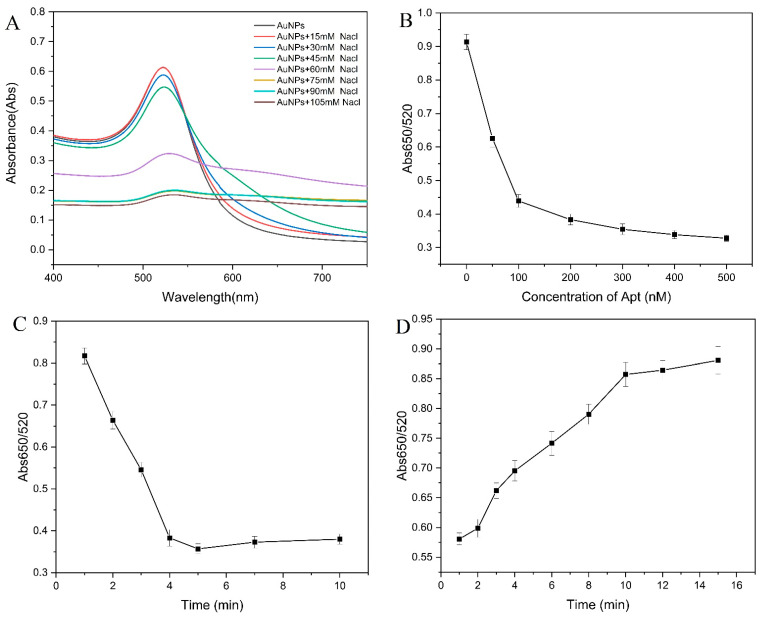
Optimization of the aptamer-AuNPs colorimetric conditions. (**A**) NaCl concentration. (**B**) Aptamer concentration. (**C**) Reaction time between AuNPs and NaCl. (**D**) Incubation time of aptamer and AuNPs.

The degree of aggregation of AuNPs is greatly affected by the concentration of NaCl. A low NaCl concentration can cause incomplete aggregation of AuNPs. A high concentration of NaCl will cause the AuNPs to accumulate too much, make the background signal too high, and interfere with the sensitivity of the experiment. Therefore, in order to ensure a wide detection range and a low detection limit, the optimization of NaCl concentration is extremely important. As the salt concentration increased in the range of 0–105 mM, the value of Abs520 continued to decrease and eventually stabilized. When the NaCl concentration was higher than 75 mM, the absorbance of the AuNPs solution at 520 nm no longer showed a significant change, indicating that the AuNPs were completely precipitated (Figure 5A). Combined with the above observation results, a NaCl concentration of 75 mM was selected for subsequent testing.

In a high NaCl concentration solution, the aptamer adsorbed on the surface of the AuNPs can protect it from the influence of NaCl, but when the concentration of the aptamer is too low, some AuNPs cannot be protected by the aptamer. However, when the aptamer concentration is too high, the AuNPs do not agglomerate in high concentrated NaCl solution, and the color does not change significantly, resulting in an increase in the detection limit. When the aptamer concentration was higher than 200 nM, the value of Abs650/520 tended to be stable (Figure 5B). This evidence indicated that the 200 nM aptamer was suitable for further experiments.

Under certain conditions, the reaction time is also an important influencing factor for the combination. In order to eliminate the error caused by the reaction time, the influence of the incubation time of AuNPs and NaCl was investigated. The results are shown in Figure 5C. When the reaction time was 10 min, the value of Abs650/520 reached the maximum. With the extension of time, the absorbance no longer changed significantly. Therefore, 10 min was selected as the detection time of the system. As can be seen from Figure 5D, as the incubation time of AuNPs and aptamer increased, the value of Abs650/520 decreased and reached the lowest value at 5 min. Therefore, 5 min was selected as the binding time of AuNPs and aptamer.

#### 3.5.3. Performance Analysis of the Aptamer-AuNPs Colorimetric Detection of AFM1

**Figure 6:**
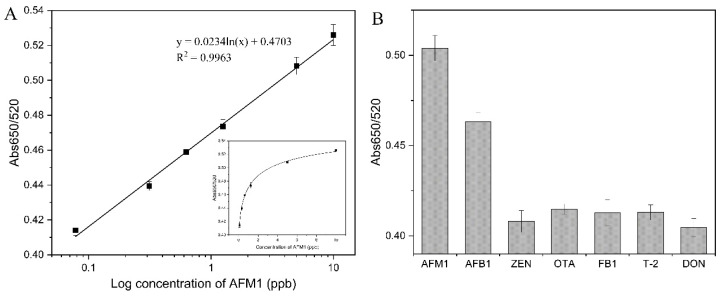
(**A**) Standard curve of the aptamer-AuNPs colorimetric method for detection of AFM1. (**B**) Specificity of aptamer-AuNPs colorimetric method for detection of AFM1.

In the aptamer-AuNPs colorimetric system, the aptamer specifically binds to AFM1, which dissociates from the surface of AuNPs; the AuNPs, after the loss of protection, are exposed to NaCl solution, aggregate, and precipitate. At the same time, the color change from red to blue can be observed with the naked eye and can be monitored by UV. As shown in Figure 6A, with the increase in AFM1 concentration, the value of Abs650/520 gradually increased, with a good linear relationship in the concentration range of 0.078–10 ng/mL. The linear regression equation was y = 0.0234lnx + 0.4703 (R^2^  =  0.9963), and the limit of actual detection was 0.078 ng/mL.

The selectivity of aptamer-AuNPs colorimetric detection of AFM1 was assessed by examining the color change of AFB1, ZEN, OTA, FB1, T-2, and DON versus AFM1 systems. Figure 6B displays the presence of even 2-fold excess (10 ng/mL) of the control targets causing insignificant color changes in the solutions, while the presence of a smaller concentration of AFM1 (5 ng/mL) resulted in a substantial increase in the colorimetric signal. Although AFB1 has a higher response than other controls, AFM1 is a metabolite of AFB1 that mainly contaminates different kinds of food. Therefore, the colorimetric assay that we constructed was able to achieve high selectivity for AFM1 detection in practical applications. The detection limit of this method was significantly improved compared to other commonly used methods and was comparable to many AFM1 complex detection strategies (Table 1).

#### 3.5.4. Real Sample Analysis

This colorimetric method was designed to detect AFM1 in milk. To study the matrix effect, we established extraction-calibrated detection curves using milk extract instead of buffer; the calibration equation obtained from this curve was y = 0.0171lnx + 0.4669 (R^2^ = 0.9959) (Figure S5). It showed that the colorimetric method we established was basically not affected by the food matrix and had strong stability. Additionally, we performed the same experimental procedure on spiked milk samples (0.1, 0.5, and 1 ng/mL) to evaluate the practical application of this method. The analysis results are summarized in Table 2. The average recovery of the developed colorimetric method ranged from 96.83% to 108.17%. Method validation was performed using a commercial ELISA kit with recoveries ranging from 92.35 to 102.97%. After analysis of variance, there was no significant difference (*p* > 0.05) between the two methods, which matched the results of our developed aptamer-AuNPs colorimetric assay, indicating that the proposed aptamer-AuNPs colorimetric method can be used as a robust and sensitive detection method for AFM1 in milk.

## 4. Conclusions

We applied the synthesized MGO nanomaterials to the real-time monitoring SELEX platform and obtained a high-affinity ssDNA aptamer of AFM1. The Kd values of the 24 selected aptamers were all at the nanomolar level with the lowest value of 8.2 nM, with a sequence homology greater than 97%. The binding sites and recognition mechanisms of AFM1 and aptamers were predicted and analyzed by molecular docking, demonstrating that several sites can bind AFM1 molecules through hydrogen bonding and hydrophobic interactions, which were consistent with the experimental analysis. Using the aptamer-AuNPs system, a label-free colorimetric method was developed and used for the detection of AFM1 in milk samples. Additionally, using a commercial ELISA kit as a comparison, the practical application of the colorimetric method in the recovery of milk samples was verified.

## Figures and Tables

**Table 1:** Comparison of the developed colorimetric method with other different detection methods for AFM1 determination.

Methods	Sensing Platform	Detection Range (ng/mL)	LOD (ng/mL)	Reference
UPLC-MS/MS	-	-	2.7	[37]
Immunochromatographic test strip	Eu (III)-TRFM	0.05–2.0	0.048	[38]
Fluorescence	Fe_3_O_4_-SPE	0.04–8	0.015	[39]
Electrochemical	Anti-idiotype nanobodies	0.25–5.0	0.09	[40]
Electrochemiluminescence	Apt-GMNPs-GO-L-AgNPs	5–150	0.01	[41]
Colorimetric assay	Apt-AuNPs	0.078–10	0.078	This work

**Table 2:** Recoveries of AFM1 in samples (*n* = 3).

Sample	AFM1 Spiked (ng/kg)	This Work		Commercial ELISA Kits	
		AFM1 Found (ng/kg)	Recovery (%)	AFM1 Found (ng/kg)	Recovery (%)
Milk	1	0.96 ± 0.07	96.83 ± 7.33	1.05 ± 0.05	102.97 ± 2.36
	0.5	0.49 ± 0.04	99.07 ± 9.06	0.46 ± 0.03	92.35 ± 7.00
	0.1	0.11 ± 0.01	108.17 ± 8.61	0.09 ± 0.01	93.25 ± 5.72

## Data Availability

The data presented in this study are available on request from the corresponding author.

## Supplementary Materials

The following supporting information can be downloaded at: https://www.mdpi.com/article/10.3390/foods11121802/s1, Table S1: Conditions for 12 rounds of MGO-SELEX screening; Table S2: Aptamer sequence and its Kd value; Figure S1: (A) TEM and (B) VSM of MGO; Comparison of the (C) mass ratio and (D) incubation time of MGO and GO to ssDNA library; Figure S2: Optimization of the binding affinity conditions; Figure S3: Affinity nonlinear fitting curve of 24 aptamers; Figure S4: UV absorption curve (A) and TEM (B) of AuNPs; Figure S5: Detection curve developed in the milk extract.
