# Supplementary material for: Selection and Characterization of DNA Aptamers for Constructing Aptamer-AuNPs Colorimetric Method for Detection of AFM1
Source: Foods. 2022 Jun 18;11(12):1802. doi: 10.3390/foods11121802 (PMC9222373; doi:10.3390/foods11121802)
Supplement: Supplementary file 1 [file foods-11-01802-s001.zip › foods-1748547-supplementary.pdf]

Selection and Characterization of DNA Aptamers for Constructing Aptamer-AuNPs Colorimetric Method for Detection of AFM1

Ruobing Liu [1], Fuyuan Zhang [1], Yaxin Sang [1], Minxuan Liu [1], Minghui Shi [1] and Xianghong Wang [1,*]

[1] College of Food Science and Technology, Hebei Agricultural University, Baoding 071001, China. Koori0520@163.com (Ruobing Liu); zhang.fuyuan@hotmail.com (Fuyuan Zhang); sangyaxin@hebau.edu.cn (Yaxin Sang); lmx0325@126.com (Minxuan Liu); 1870612098@qq.com (Minghui Shi).

* Correspondence: wangshipin2017@163.com (Xianghong Wang).

Supplementary Materials:

Table S1. Conditions for 12 rounds of MGO-SELEX screening

SELEX Round	ssDNA Added (pmol)	Time (min)	Counter SELEX	Recovery
1	1000	120	N	10.08%
2	500	120	N	12.33%
3	500	120	N	15.61%
4	200	100	N	22.94%
5	200	100	N	26.15%
6	100	80	ZEN(100pmol)	30.38%
7	100	80	OTA(100pmol)	36.59%
8	100	80	FB1(100pmol)	42.37%
9	100	80	DON(100pmol)	47.86%
10	100	80	T-2 (100pmol)	50.70%
11	50	60	N	52.71%
12	50	60	N	52.33%

Table S2. Aptamer sequence and its Kd value

Apt	Squence (5’-3’)	Length (nt)	Kd (nmol/L)
1	AGCAGCACAGAGGTCAGATGGGATGTGAGGTGGCTCTCGTATCTATAGATTTTGACGAGACCTATGCGTGCTACCGTGAA	80	19.02±1.939
2	AGCAGCACAGAGGTCAGATGAGGTACTTGTGCCGCGTTAATCGAGTGCTTTGCTCTCGGACCTATGCGTGCTACCGTGAA	80	30.90±2.711
3	AGCAGCACAGAGGTCAGATGGGATGTGAGGTGGCTCTCGTATCTATAGATTTTGACGAGGCCTATGCGTGCTACCGTGAA	80	26.95±4.133
4	AGCAGCACAGAGGTCAGATGTCGTCTCACGACTTCTTTCTTGCGCTGTCGGCCGAGAAACCCTATGCGTGCTACCGTGAA	80	12.15±1.91
5	AGCAGCACAGAGGTCAGATGGGATGTGAGGTGGCTCTCGTATCTATAGATTTTGGCGAGACCTATGCGTGCTACCGTGAA	80	8.12±1.509
6	AGCAGCACAGAGGTCAGATGGGATGTGAGGTGGCCCTCGTATCTATAGATTTTGACGAGACCTATGCGTGCTACCGTGAA	80	12.44±2.74
7	AGCAGCACAGAGGTCAGATGGGATGTGGGGTGGCTCTCGTATCTATAGATTTTGACGAGACCTATGCGTGCTACCGTGAA	80	11.71±3.795
8	AGCAGCACAGAGGTCAGATGAGCCAGGGGCGGCGCAAATTTTCCGCTAAAATTTGAAGACCCTATGCGTGCTACCGTGAA	80	16.80±3.32
9	AGCAGCACAGAGGTCAGATGGGATGTGAGGTGGCTCCCGTATCTATAGATTTTGACGAGACCTATGCGTGCTACCGTGAA	80	14.53±3.08
10	AGCAGCACAGAGGTCAGATGGGACGTGAGGTGGCTCTCGTATCTATAGATTTTGACGAGACCTATGCGTGCTACCGTGAA	80	8.78±1.713
11	AGCAGCACAGAGGTCAGATGGGATGTGAGGCGGCTCTCGTATCTATAGATTTTGACGAGACCTATGCGTGCTACCGTGAA	80	14.53±2.47
12	AGCAGCACAGAGGTCAGATGGGATGTGAGGTGGCTCTCGTATCTATAGATGTTGACGAGACCTATGCGTGCTACCGTGAA	80	27.07±3.52
13	AGCAGCACAGAGGTCAGATGTGTCTGAGGGGTACCCTTTCGTTGACTTTGTCGACAAACTCCTATGCGTGCTACCGTGAA	80	35.62±4.392
14	AGCAGCACAGAGGTCAGATGGGATGCGAGGTGGCTCTCGTATCTATAGATTTTGACGAGACCTATGCGTGCTACCGTGAA	80	19.05±2.61
15	AGCAGCACAGAGGTCAGATGGGATGTGAGGTGACTCTCGTATCTATAGATTTTGACGAGACCTATGCGTGCTACCGTGAA	80	22.06±3.29
16	AGCAGCACAGAGGTCAGATGCGCGGAGACCGTTTGGTCGGAGCGTTTGCATTGACGAAAACCTATGCGTGCTACCGTGAA	80	20.12±3.011
17	AGCAGCACAGAGGTCAGATGGGATGTGAGGTGGCTCTCGTACCTATAGATTTTGACGAGACCTATGCGTGCTACCGTGAA	80	13.86±2.485
18	AGCAGCACAGAGGTCAGATGGGATGTGAGGTGGCTCTCGTATCTATAGACTTTGACGAGACCTATGCGTGCTACCGTGAA	80	13.87±2.57
19	AGCAGCACAGAGGTCAGATGGGATGTGAGGTGGCTCTCGTATCTATAGATTTTGACGGGACCTATGCGTGCTACCGTGAA	80	18.75±2.085
20	AGCAGCACAGAGGTCAGATGGGATGTGAGGTGGCTCTCGCATCTATAGATTTTGACGAGACCTATGCGTGCTACCGTGAA	80	30.73±3.80
21	AGCAGCACAGAGGTCAGATGGGGTGTGAGGTGGCTCTCGTATCTATAGATTTTGACGAGACCTATGCGTGCTACCGTGAA	80	16.47±3.39
22	AGCAGCACAGAGGTCAGATGGGATGTGAGGTGGCTCTCGTATCTATGGATTTTGACGAGACCTATGCGTGCTACCGTGAA	80	19.12±3.11
23	AGCAGCACAGAGGTCAGATGGGATGTGAGGTGGCTCTCGTATCTACAGATTTTGACGAGACCTATGCGTGCTACCGTGAA	80	18.01±3.555
24	AGCAGCACAGAGGTCAGATGGGATGTGAGGTGGCTCTCGTGTCTATAGATTTTGACGAGACCTATGCGTGCTACCGTGAA	80	18.64±3.24
5T1	AGGTCAGATGGGATGTGAGGTGGCTCTCGTATCTATAGATTTTGGCGAGACCT	53	10.53±1.59
5T2	GGGATGTGAGGTGGCTCTCGT	21	10.04±1.34
5M	AGCAGCACAGAGGTCAGATGGGATGTCCGGTGGCTCCCGTATCTATAGATTTTGGCGAGACCTATGCGTGCTACCGTGAA	80	-

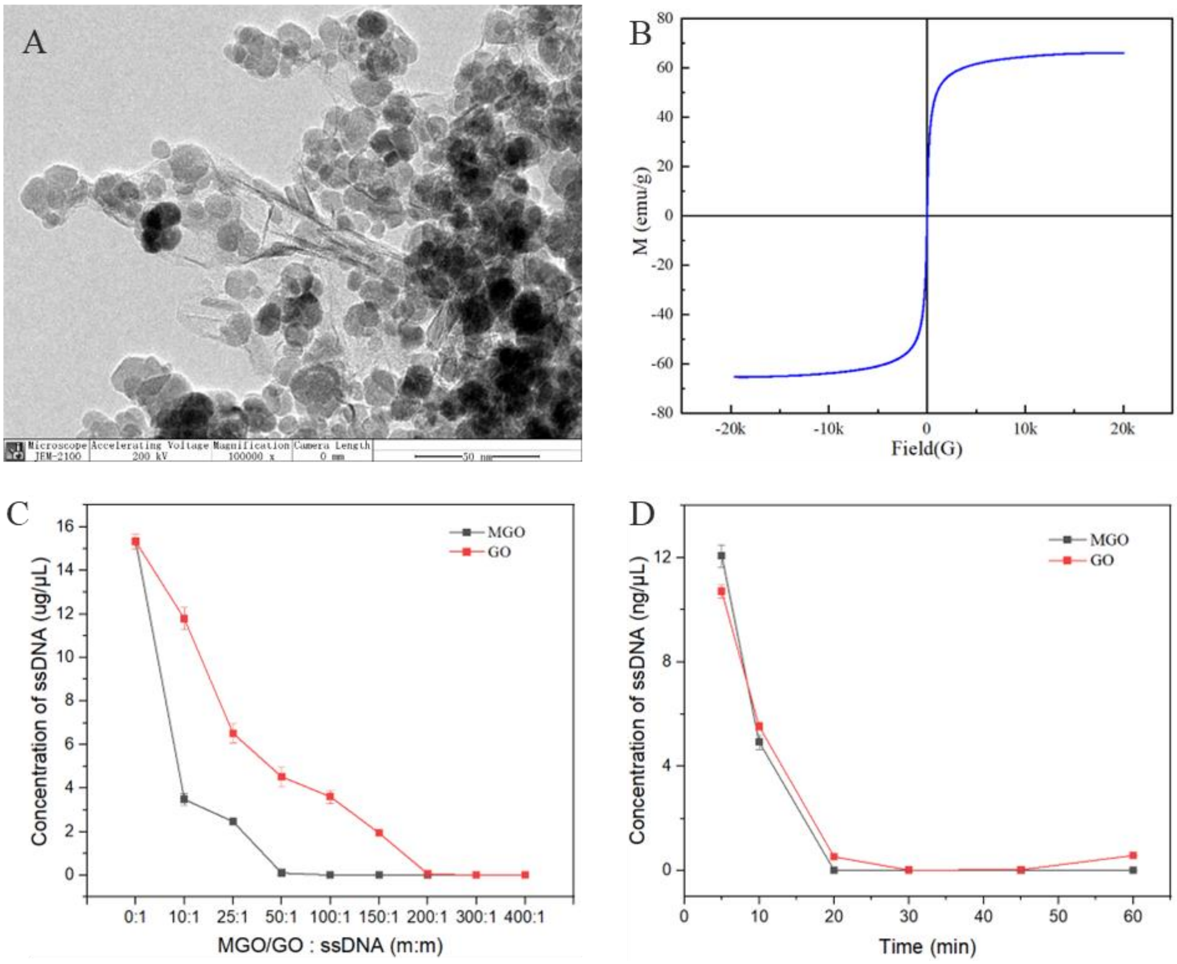

Figure S1. (A)TEM and (B)VSM of MGO, Comparison of the (C) mass ratio and (D) incubation time of MGO and GO to ssDNA library.

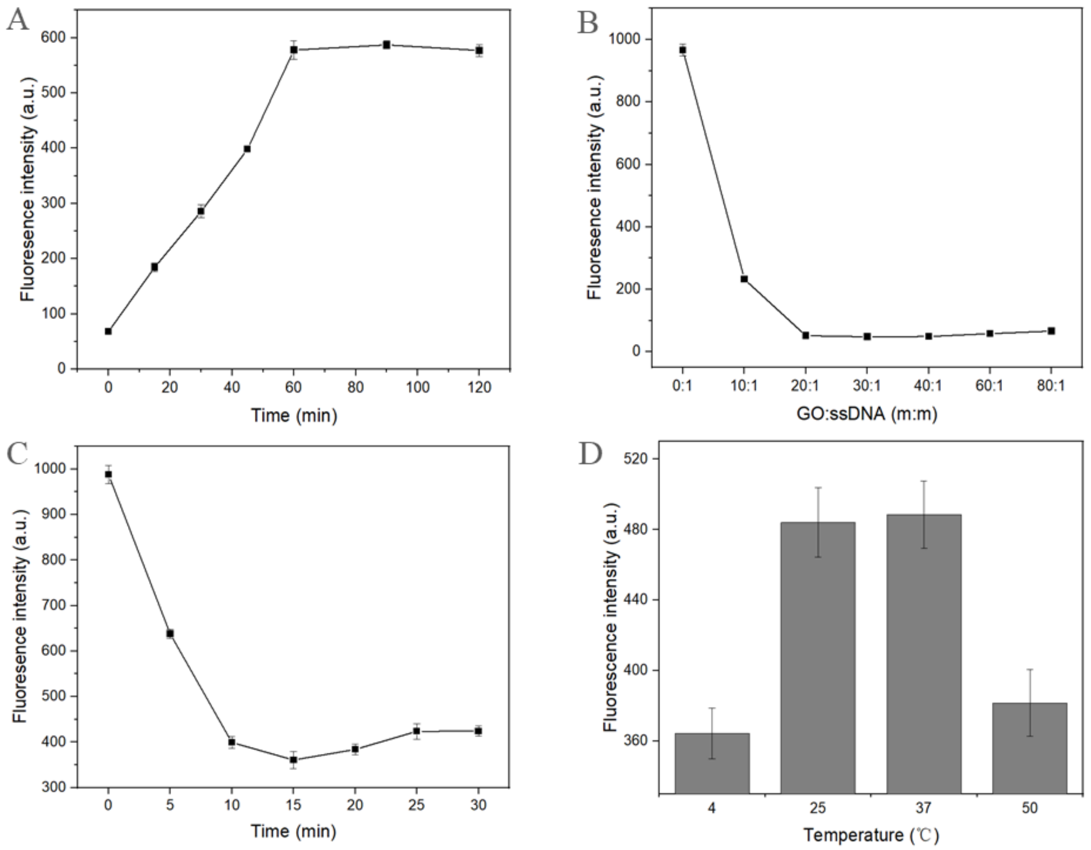

Figure S2. Optimization of the binding affinity conditions. (A) Binding time of aptamer and AFM1, (B) Mass ratio of GO and FAM-labeled aptamers, (C) Quenching time, (D) Working temperature.

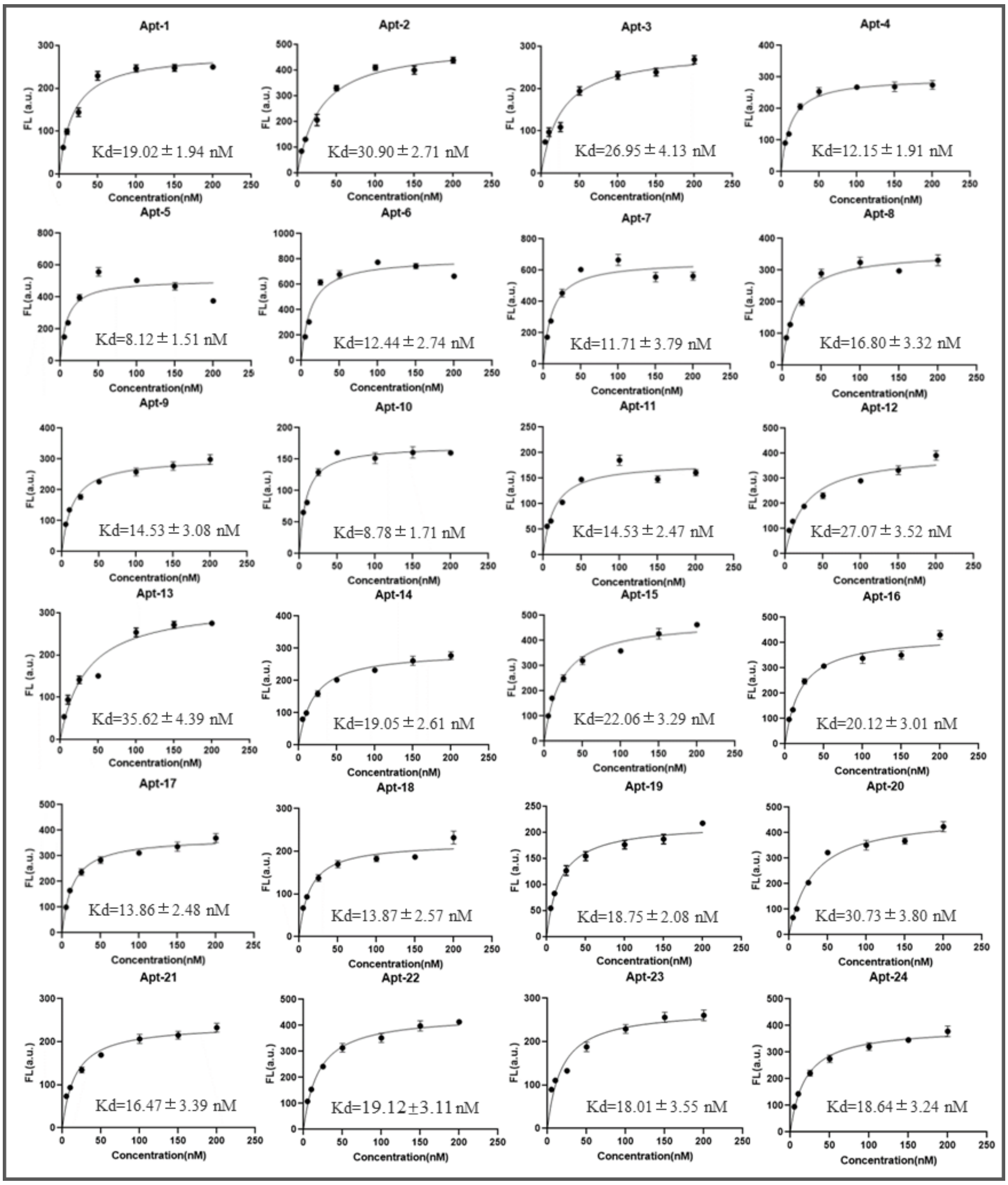

Figure S3. Affinity nonlinear fitting curve of 24 aptamers.

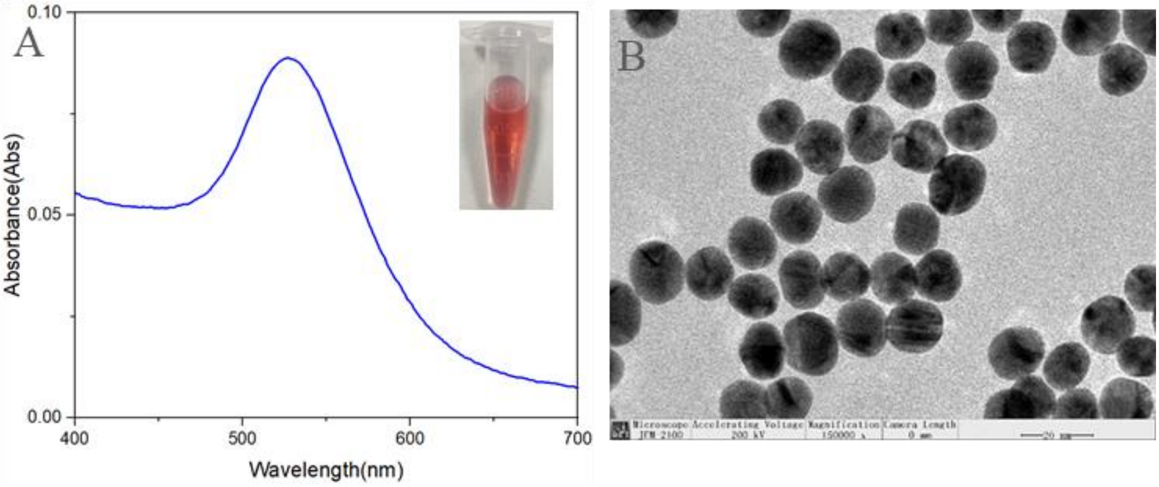

Figure S4. UV absorption curve (A) and TEM (B) of AuNPs.

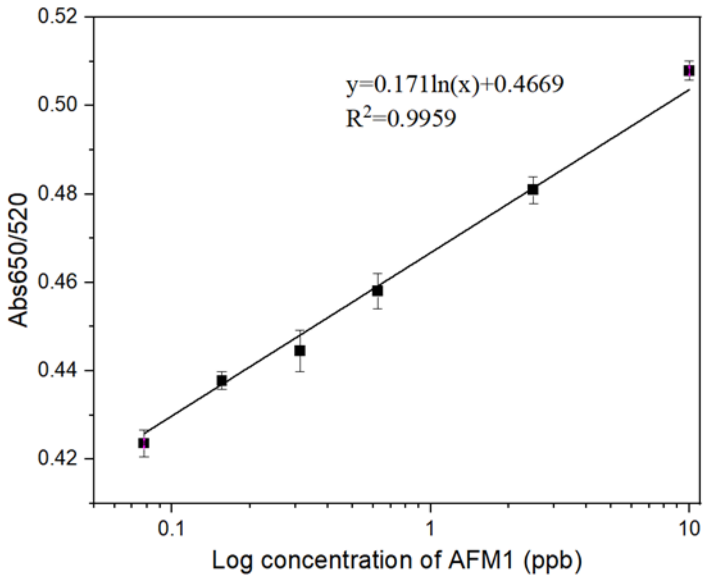

Figure S5. Detection curve developed in the milk extract.
